# Bioactive Potential: A Pharmacognostic Definition through the Screening of Four *Hypericum* Species from the Canary Islands

**DOI:** 10.3390/molecules27186101

**Published:** 2022-09-18

**Authors:** Rodney Lacret, Adrián Puerta, Sebastian Granica, Aday González-Bakker, Danela Hevia, Yiling Teng, Candelaria C. Sánchez-Mateo, Pedro Luis Pérez de Paz, José M. Padrón

**Affiliations:** 1 BioLab, Instituto Universitario de Bio-Orgánica Antonio González (IUBO-AG), Universidad de La Laguna, Avda. Astrofísico Francisco Sánchez 2, 38206 La Laguna, Spain; 2 Departamento de Medicina Física y Farmacología, Facultad de Farmacia, Universidad de La Laguna, Tenerife, 38200 La Laguna, Spain; 3 Microbiota Lab, Centre of Preclinical Studies, Medical University of Warsaw, Banacha 1b, 02-097 Warsaw, Poland; 4 Departamento de Botánica, Ecología y Fisiología Vegetal, Facultad de Farmacia, Universidad de La Laguna, Tenerife, 38200 La Laguna, Spain

**Keywords:** bioactive potential, anticancer potential, total activity, screening, *Hypericum canariense*, *Hypericum glandulosum*, *Hypericum grandifolium*, *Hypericum reflexum*

## Abstract

In this work, we propose a general methodology to assess the bioactive potential (BP) of extracts in the quest of vegetable-based drugs. To exemplify the method, we studied the anticancer potential (AP) of four endemic species of genus *Hypericum* (*Hypericum canariense* L, *Hypericum glandulosum* Aiton, *Hypericum grandifolium* Choisy and *Hypericum reflexum* L.f) from the Canary Islands. Microextracts were obtained from the aerial parts of these species and were tested against six human tumor cell lines, A549 (non-small-cell lung), HBL-100 (breast), HeLa (cervix), SW1573 (non-small-cell lung), T-47D (breast) and WiDr (colon). The methanol–water microextracts were evaluated further for cell migration, autophagy and cell death. The most promising bioactive polar microextracts were analyzed by UHPLC–DAD–MS. The extraction yield, the bioactivity evaluation and the chemical profiling by LC–MS suggested that *H. grandifolium* was the species with the highest AP. Label-free live-cell imaging studies on HeLa cells exposed to the methanol–water microextract of *H. grandifolium* enabled observing cell death and several apoptotic hallmarks. Overall, this study allows us to select *Hypericum grandifolium* Choisy as a source of new chemical entities with a potential interest for cancer treatment.

## 1. Introduction

The chemical bioprospecting of new therapeutic agents, pesticides, nutraceuticals, and other products with biotechnological applications from plants is one of research lines with the greatest impact worldwide [1,2,3]. The chemical bioprospecting protocol includes (a) the selection and extraction of appropriate plants, (b) the evaluation of the bioactive potential of the extracts to select the species with the highest bioactive potential, and (c) the bioguided isolation of compounds from the best candidates [4]. Thus, the rationalization of each stage of the chemical bioprospecting contributes to accelerate the discovery of compounds with high added value [5,6].

Bioactive potential (BP) is a very often employed term in the search of bioactive compounds from living organisms (bacteria, fungi or plants) but its definition is not clear yet in the literature. BP can be subdivided into agrochemical or pharmacological potential depending on the field of the research, i.e., agrochemistry or pharmacology, respectively [3,7]. In drug discovery, the bioactive potential of extract from plants as a source of high-value compounds has mainly focused the bioactivity evaluation and dereplication of extracts [8,9,10]. Eloff and coworkers introduced a parameter called total activity, a mathematical relationship between extraction yield and the antimicrobial activity for the quantification of the bioactivity of plan extracts during the screening [11]. However, there are a few examples in the literature that use the total activity as a tool [12,13,14]. On the other hand, rules or recommendations of thumb for defining anti-infective potential in natural products were developed by Cos and coworkers [15]. It is worthwhile to mention that these recommendations did not take into account the total activity previously reported and they are only related to the anti-infective activity. From this state of art, we propose to define bioactive potential as a combination of three parameters, namely, the extraction process, the biological activity profiling (either agrochemical or pharmacological) and the early chemical profiling. The main goal of the evaluation of BP is to prioritize, through extracts from plants, the rational selection of the best vegetable species for the discovery of new chemical bioactive entities. BP can be classified into different types depending on the field of bioassays (antibacterial, anti-inflammatory, anticancer, biocidal, etc.). BP can be also applied to the bioguided isolation process.

The genus *Hypericum* (Hypericacea) comprises high-value medicinal plants, particularly *Hypericum perforatum* L., which is largely used in folk medicine [16]. Extensive pharmacological and chemical studies on the genus *Hypericum* have revealed its potential as a source of antibacterial, anti-inflammatory, antiproliferative and antiviral drugs [17,18,19,20,21,22]. In the Canary Islands, the genus *Hypericum* is comprised of eight species: *H. canariense*, *H. coadunatum*, *H. glandulosum*, *H. grandifolium*, *H. humifusum*, *H. perfoliatum*, *H. perforatum* and *H. reflexum*. Likewise, the aerial parts of most of them have been used in the traditional medicine as vulnerary, sedative, tranquilizer, vermifuge, diuretic, tinctorial and as food for goats [23,24,25]. The chemical and pharmacological studies on these *Hypericum* species from the Canary Islands have been conducted for more than 35 years [26,27,28,29,30,31,32,33,34,35,36,37,38,39,40,41,42]. From them, eleven works have been published related to the evaluation of pharmacological actitivities of extracts from *H. canariense*, *H. glandulosum*, *H. grandifolium* and *H. reflexum* [26,27,28,29,30,31,32,33,34,35,42] and six papers have adressed the isolation of compounds from *H. canariense*, *H. coadunatum* and *H. reflexum* [36,37,38,39,40,41]. Nevertheless, to date, there are no bioguided chemical studies on these *Hypericum* species from the Canary Islands that provide information about compounds responsible for the previous observed pharmacological activities.

Since 2019, our research interests focusses on the chemical bioprospecting of plants from the Canary Islands as a source of high-added-value compounds and the evaluation of their BP with a particular interest in the anticancer potential (AP). The aim of our approach is to identify the plants with the highest AP. In this step, the extraction of plants at a small scale (microextraction), the biological activity profiling of these microextracts, and their early chemical profiling by LC–MS are considered relevant factors as a whole. The outcome of this selection process is a sorted (prioritized) list of plants from the highest to the lowest AP. In this work, we will show the implementation of our strategy with the evaluation of the AP of four *Hypericum* species from the Canary Islands, namely, *H. canariense* L, *H. glandulosum* Aiton, *H. grandifolium* Choisy and *H. reflexum* L.f. Notably, this is the first time that these four species of *Hypericum* were profiled together both at the pharmacological and at the chemical level.

## 2. Results and Discussion

Our evaluation protocol of the AP of plants consists of three sequential steps. Every step provides relevant information to help in the assessment of the AP. In the next Sections, we will present our implementation of the methodology and we will discuss the results and the criteria we followed to prioritize the *Hypericum* species from the Canary Islands as a source of bioactive compounds.

### 2.1. Selection of Plants and Microextraction

**Figure 1:**
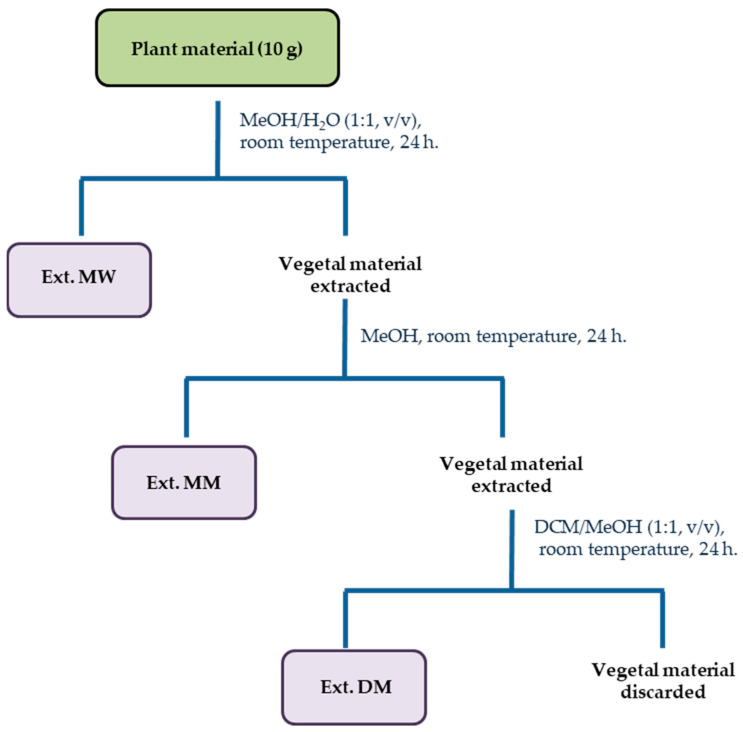
Workflow procedure for the preparation of microextracts from aerial parts of *Hypericum* species from the Canary Islands.

*H. canariense*, *H. glandulosum*, *H. grandifolium* and *H. reflexum* were selected considering chemosystematic and ethnopharmacological approaches [7]. The extraction process of these species started with the maceration of their aerial part at a small scale (microextraction). For this purpose, to perform the extraction in a sequential maceration, we selected as solvents water, methanol and dichloromethane. The procedure we followed is an adapted methodology previously described by Macías in the search of phytotoxic compounds [43]. This methodology was designed regarding the vast range of specialized metabolites in the *Hypericum* genus (range of polarities and bioactivities) [22,44] and as a way to solve the difficulty to obtain them using a single extraction [45]. Therefore, each plant dried material (approximately 10 g) was sequentially macerated under the same conditions from polar to non polar solvents considering a sample ratio of 20:1 (v/w). Firstly, a methanol/water (1:1) mixture (MW), then methanol (MM), and finally a dichloromethane/methanol (1:1) mixture (DM) were used, as depicted in Figure 1.

After the extraction process, three microextracts from each plant were obtained (MW, MM and DM). Thus, a total of 12 microextracts were prepared. For each microextract, the dry mass was weighed and the extraction yield, expressed in percentage, was calculated. The results are shown in Table 1.

The extraction yield is a pharmacognostic parameter used for considering any vegetal as a source of leads or drugs [14]. It also enables the calculation of the total activity (TA) (Equation (1), ), a useful tool for the comparison of crude extracts during screening of plants as a source of bioactive compounds [11]. In our study, the MW and MM microextracts from the aerial parts of the four *Hypericum* species gave the highest yields. In contrast, the DM microextracts provided the lowest yields, except for *H. glandulosum*. In general, these data suggested that *Hypericum* species from the Canary Islands were rich in medium and polar compounds. These results also match with a recently published study on *Hypericum* species from the UK [14]. These 12 microextracts were submitted to the biological evaluation at two levels.

### 2.2. Biological Evaluation

The scientific literature holds several examples of non-mammalian and mammalian cell-based assays that have been used for the identification and characterization of bioactive natural products [46]. In the therapeutic area of cancer, screenings based on mammalian cell cultures have enabled the rapid study of a multitude of natural product extracts. In drug discovery programs of plant extracts (such as the NCI’s), the first biological assessment is an antiproliferative test against tumor cell lines (a general or primary bioassay) [47,48].

#### 2.2.1. Antiproliferative Activity

**Figure 2:**
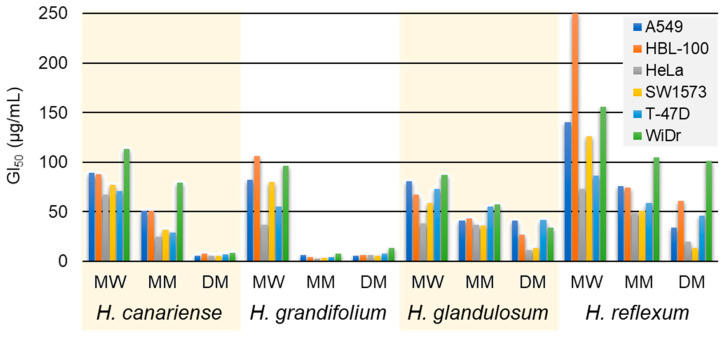
Antiproliferative activity (GI_50_) of microextracts from *Hypericum* species. MW = methanol–water; MM = methanol; DM = dichloromethane–methanol.

Following the guidelines of the NCI, the antiproliferative activity of the 12 microextracts (MW, MM and DM) obtained from the aerial parts of *H. canariense* (CMW, CMM and CDM), *H. glandulosum* (GMW, GMM and GDM), *H. grandifolium* (LMW, LMM and LDM), and *H. reflexum* (RMW, RMM and RDM) were evaluated against six human tumor cell lines: A549, HBL-100, HeLa, SW1573, T-47D and WiDr. The maximum test concentration was set at 250 µg/mL, according to NCI guidelines [49,50]. The results, expressed as 50% growth inhibition (GI_50_), are given in Figure 2 (Table S1). According to NCI calculations, GI_50_ values represent approximate values. Taken as a whole, the GI_50_ values enabled an arrangement of the plants in the following decreasing order of activity: *H. grandifolium* > *H. canariense* > *H. glandulosum* > *H. reflexum*. Our results are in agreement with a previous study where the antiproliferative activity of essential oils and polar extracts (methanol—acetone 1:1) from the aerial parts of *H. canariense*, *H. grandifolium* and *H. reflexum* were evaluated against the human tumor cell lines A375 (malignant melanoma), MDA-MB-231 (breast adenocarcinoma) and HCT116 (colon carcinoma) [42]. In that study, the polar extract from *H. grandifolium* also exhibited a significant antiproliferative activity and resulted the most active one. Noteworthy, our work represents the first study reporting antiproliferative activity of *H. glandulosum* extracts. 

When we are considering the solvent extraction, the sequence obtained (in decreasing activity) for our samples was DM > MM > MW. In the particular case of *H. grandifolium*, the DM and MM microextracts (i.e., GDM and GMM, respectively) were the most active ones against all the tumor cell lines tested. They displayed the lowest GI_50_ values (in the range 2.7–13 µg/mL) among the twelve assayed microextracts (Figure 2). However, these GI_50_ values denote a highly cytotoxic profile (GI_50_ ≤  20 µg/mL) according to the NCI’s guidelines [50]. Noteworthy, the MW microextracts induced moderate cytotoxicity (GI_50_ 21–200 µg/mL). In our experience, highly cytotoxic samples induce large cell killing and biological effects are more difficult to be observed. In contrast, samples with moderate cytotoxicity allow to measure relevant phenotypic changes in treated cells. According to this criterion and our interest in the pharmacognostic study of polar extracts, the hydroalcoholic microextracts were the prioritized for further testing.

#### 2.2.2. Total Activity

The TA (Equation (1)) is a significant pharmacognostic parameter for the quantification of the antimicrobial activity of plant extracts and it is very useful in the rational screening of diverse plants. This tool has been used as a part of the evaluation of the antibacterial activity of extracts from south African species [12]. TA, expressed in (mL/g), was defined as “the largest volume to which the bioactive compounds in one gram of plant material can be diluted and still inhibit the growth of the tested organisms” [11]. Analogously, we have envisioned the calculation of the TA when considering the antiproliferative activity of plant extracts against human solid tumor cells. From the pharmacological point of view, the growth inhibition (GI_50_ in µg/mL) indicates the potency of extracts, while TA represents the efficacy of the extracts [11].

(1) $$TA\;\left(\frac{mL}{g}\right)=\frac{m_{extract}\;\left(mg\right)}{m_{plant}\;\left(g\right)}\times \frac{1}{GI_{50}\;\left(\mu g/mL\right)}$$

In this work, we calculated the TA of the *Hypericum* species to compare the antiproliferative efficacy of their microextracts. To the best of our knowledge, this is the first time that the TA is reported to assess the antiproliferative activity during the screening of the of plant species.

**Figure 3:**
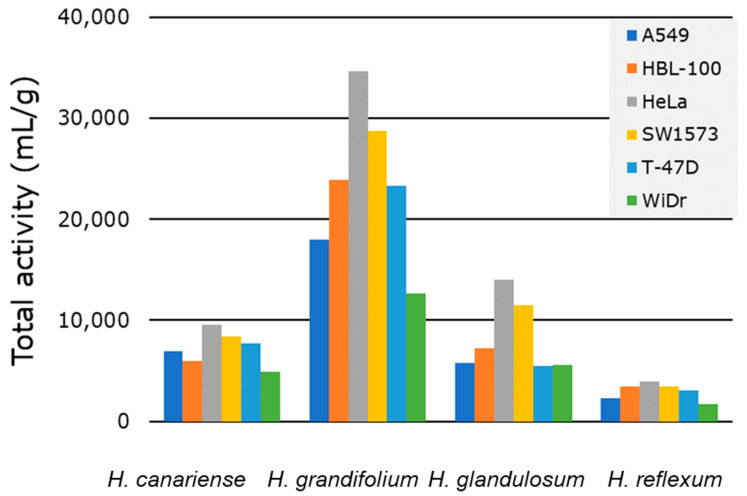
Total activity (GI_50_) of microextracts from *Hypericum* species.

Since our protocol involves the sequential extraction using three solvent systems (Figure 1), we considered appropriate to compute the TA for each individual extract and sum all the TAs corresponding to a virtual total extract from each plant and each cell line. With this calculation, each TA value represents a theoretical activity of each theorical total extract against each cell line that complies with the definition provided by Eloff [11]. Therefore, Figure 3 (Table S2) collects the TA for each of the plant species against each of the six cell lines tested. The results enabled ordering the species according to decreasing values of TA: *H. grandifolium* > *H. glandulosum* > *H. canariense* > *H. reflexum*.

Based on the GI_50_ data (Figure 2), the TA values (Figure 3 and Table S2) and our interest in polar extracts, we selected for further studies the MW microextracts from *H. canariense* (CMW)*, H. glandulosum* (LMW) and *H. grandifolium* (GMW). The sibling microextract of *H. reflexum* (i.e., RMW) were also tested for comparison purposes. At this point, many different phenotypic experiments can be considered to depict the overall mode of action of the microextracts and give information about possible cell targets. These experiments are generally termed as secondary bioassays. The literature reports diverse secondary bioassays for the potential anticancer activity of *Hypericum* sp. extracts [19,20,21]. Herein, we selected as secondary tests cell migration and vacuole formation, two relevant hallmarks for cancer treatment, which have not been reported for the (four) *Hypericum* sp. (included in this work). Cell migration experiments give an idea of the antimetastatic potential of the tested samples [51], while vacuole formation is an indicator of autophagy [52] and drug resistance mechanisms [53], among others.

#### 2.2.3. Cell Migration Disturbances in A549 Cells

Cell migration is the ability of a cancer cell to undergo movement and invasion allowing it to relocate within the tissues [51]. This process allows neoplastic cells to enter lymphatic and blood vessels for dissemination into the circulation metastasize in distant organs. The non-small-cell lung cancer (NSCLC) cell line A549 is considered as a highly metastatic cancer cell line [54] and represents a good in vitro model to assess the antimigratory properties using the wound healing assay [55].

**Figure 4:**
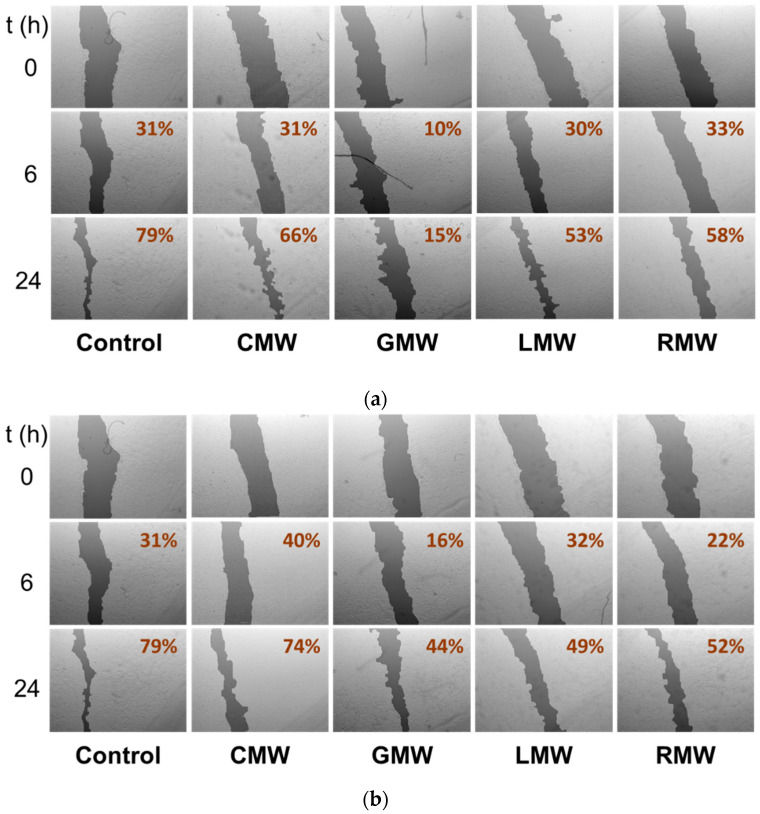
Wound closure assay results on A549 cells when using 100 µg/mL (**a**) and 50 µg/mL (**b**) of MW microextracts. Wound closure percentage with respect to time = 0 is given on the right top corner of each image.

To study the effects on A549 cell migration of the MW microextracts of the four *Hypericum* species, we selected two doses, a high (100 µg/mL) and a low dose (50 µg/mL), and two time points, 6 and 24 h (Figure 4). The use of this early time point enables avoiding, at minimum, the effect of cell proliferation in the assay, limiting the response to the antimigratory activity of the microextract. With the exception of CMW all microextracts, were able to reduce the closure of the wound when compared to control wells after 6 and 24 h. GMW was able to inhibit cell migration after six hours of treatment in a concentration dependent manner, being the most active of all microextracts assayed. Interestingly, CMW, which showed antiproliferative activity, failed to reduce the migration of A549 cells. Meanwhile, both LMW and RMW did not show correlation between dose and inhibition of cell mobility.

#### 2.2.4. Formation of Acidic Vacuoles in HeLa Cells

**Figure 5:**
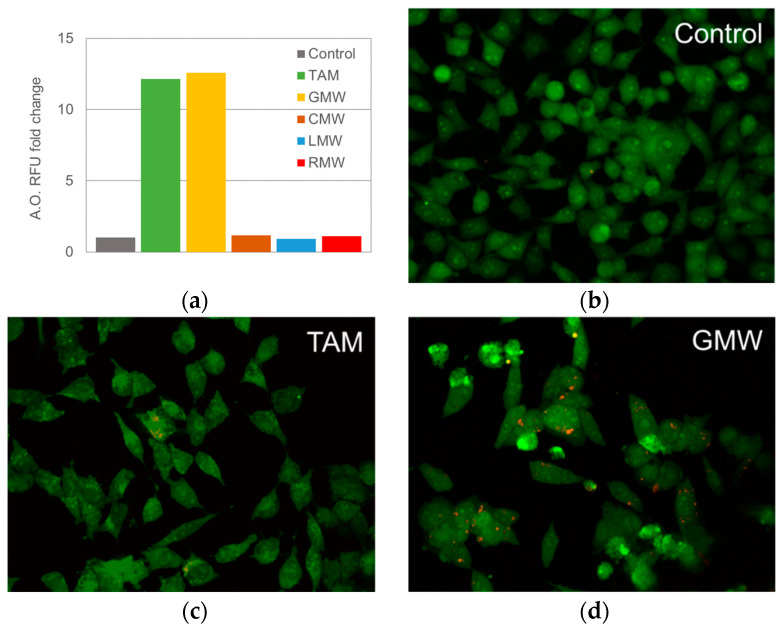
(**a**) Relative fluorescence units (RFU) compared to control. HeLa cells were treated for 24 h with 100 µg/mL of each extract. (**b**–**d**). Representative images of fluorescence microscopy after 24 h of incubation with 10 µM of TAM and 100 µg/mL of GMW. Captured images in green and red were merged using ImageJ.

Considering the antiproliferative profile of the *Hypericum* extracts, CMW, GMW, LMW and RMW were selected to perform acridine orange (AO) staining in order to evaluate the formation of acidic vacuoles in the cells after the treatment. AO is a fluorescent dye that binds to acidic compartments. Differences in fluorescence intensity could represent changes in cell response to the plant microextract, of particular interest to the formation of acidic vacuoles [56]. As a positive control we used 10 µM of tamoxifen (TAM), an established anticancer drug whose ability to enhance cell vacuolization has been reported previously [57]. As depicted in Figure 5a, GMW showed the highest fold change in RFU over control, similar to the effect produced by TAM, whilst the rest of the microextracts did not produce a representative fold change when treated with 100 µg/mL for 24 h. Taking this into account and considering the previous result, we proceeded to evaluate GMW microextract effects on HeLa cells under fluorescence microscopy. Images taken agreed with the data obtained by spectrofluorimetric assays, with this microextract enhancing the accumulation of the dye inside clearly visible vacuoles (Figure 5b).The accumulation of AO inside acidic vacuoles produces a metachromatic shift from green to red fluorescence [58].

To sum, the biological tests (primary and secondary bioassays) of the *Hypericum* species resembled the aforementioned order based on the TA values. Thus, the relevance of the plants was established as *H. grandifolium* > *H. glandulosum* > *H. canariense* > *H. reflexum*. The lower TA values together with neglected effects observed in the biological tests (Figure 4 and Figure 5) allow us do not consider the MW microextract *H. reflexum* for the chemical analysis by LC–MS. Accordingly, the MW microextracts of *H. canariense*, *H. glandulosum* and *H. grandifolium* were submitted to the study of their chemical profiling. Overall, the results might anticipate a different chemical profile of these microextracts.

### 2.3. Early Chemical Profiling of MW Microextracts 

The final step for the evaluation of the anticancer potential was to analyze the chemical composition of the selected bioactive MW microextracts from the aerial parts of *H. canariense*, *H. glandulosum* and *H. grandifolium* (i.e., CMW, LMW and GMW, respectively). In an effort to estimate their chemical qualitative composition in terms of specialized metabolites, they were analyzed by UHPLC–DAD–MS (Table 2). Methanol and acetone–methanol extracts from *H. canariense* and *H. grandifolium* were analyzed by LC–MS and reported previously [33,42]. This work represents the first preliminary chemical study of *H. glandulosum* extract.

Table 2 shows the chemical profiles of the three *Hypericum* MW microextracts at 280 nm. These chromatograms also show the UHPLC fingerprint of these species and the substantial differences in their chemical composition, both in terms of the number of peaks and in the proportion of common peaks (Figure S1). We speculate that this differential profile is responsible for the differences observed in the biological activity of the microextracts (Figure 2, Figure 4 and Figure 5).

According to these preliminary results, a total of forty one main compounds in terms of specialized metabolites were detected in the methanol–water microextracts CMW, GMW and LMW, respectively. Twenty four were known compounds tentatively identified based on their retention times, spectral properties (UV-Vis and MS/MS patterns) and from the comparison of literature. These known compounds belonged to the family of phenolic secondary metabolites and they have previously been described or isolated from *Hypericum* extracts [19,42,60,62,63,64]. Among these known compounds **6**, **9**, **14**, **15**, **19**, **20**, **28** and **33** were ubiquitous in all MW microextracts. A careful analysis of the UV trace at 280 nm of CMW, GMW and LMW microextracts enabled the detection of at least the presence of eighteen, nineteen and twenty seven specialized metabolites, respectively. Additionally, these MW microextracts also contained a total of fifteen unidentified compounds whose spectral data suggested their novelty as natural products from *Hypericum* species since they were not found in the literature. 

In the case of CMW microextract (from *H. canariense*), six major compounds were detected and identified as chlorogenic acid isomers (**6** and **11**), mangiferin isomers (**16**), quercetin-3-*O*-glucouronide (**24**) and quercetin (**28**). Only two detected compounds in CMW (**4** and **25**) were not associated to a described natural product previous detected in *Hypericum* species. Following the same analysis for GMW, we found three outstanding peaks, including *p*-coumaroylquinic acid isomer (**19**), quercetin 3-*O*-rhamnoside (**327**) and quercetin (**28**). The chromatogram obtained for GMW (from *H. grandifolium*), showed the presence of several minor compounds, four of them (**5**, **18**, **31** and **32**) were not possible to identify it, considering their UV spectra and their MS/MS fragmentations. Likewise the analysis of LMW microextract (*H. glandulosum*) by LC–MS revealed the presence of twenty seven compounds, including seven major compounds, among them, chlorogenic acid isomers (**6**, **11** and **12**), catechin (**15**), rutin (**22)**, kaempferol -3-O-rhamnoglucoside(**26**) and an unidentified component (**40**). This analysis also enables detecting another ten unidentified compounds (**3**, **7**, **32**, **34**–**39** and **41**). These unidentified secondary metabolites in LMW are mostly less polar compounds. This microextract is also characterized by the presence of several minor compounds.

In spite of this preliminary analysis by UHPLC–DAD–MS of MW microextracts from *H. canariense*, *H. glandulosum* and *H. grandifolium*, several minor detected compounds cannot be identified due to many of them being co-eluted with major compounds. In this framework, further bioguided isolation studies might be required to clarify their identity.

### 2.4. Bioactive and Anticancer Potential

In this work, we discussed the microextraction with three different solvents, the evaluation of bioactivity of the obtained microextracts, and the chemical analysis of the most polar microextracts from the aerial parts of four *Hypericum* species from Canary Islands. These experiments might address the selection of the species with the highest bioactive potential. Therefore, we propose an empirical equation for the calculation of the bioactive potential (BP, Equation (2)) based on the previous works by Eloff and Cos [11,15], our experience on the screening of plants [65,66] and the results described in previous sections. Basically, BP and TA have similar definitions, but BP refers to the efficacy of the plant material in several related bioassays and the possibility to find new compounds as the responsible of the initial observed biological activity.


(2) (BP)=(N+1) × Total Activity  × Chemical novelty

(3) $$Chemical\;novelty=\frac{total\;number\;of\;detected\;compounds}{number\;of\;known\;compounds}\;$$

(4) $$\left(BP\right)=\left(N+1\right)\;\times \;Yield\;\;extraction\;\times \;\frac{1}{Bioactivity}\;\;\times \;Chemical\;novelty$$

In summary, the calculation of BP involves three experimental components (Equation (4)): the extraction process (yield extraction), the bioactivity evaluation (bioactivity in primary bioassays), and the chemical analysis by LC–MS (chemical novelty, Equation (3)); and a fourth factor defined as *N* + 1, where *N* means how many times a sample becomes the most active one in a bioassay. This fourth factor (*N* + 1) implies that BP never could be zero since an extract might be bioactive in any sort of biological test (intrinsic bioactivity). Therefore, after applying Equation (4), the resulting value of BP enables the sorting and prioritizing a list of plants from the highest to the lowest BP during a screening. agrochemical or pharmacological studies) related to the search of high-value products from plants. 

To exemplify the calculation of BP, considering our special interest in the anticancer activity in the polar extracts from of the aerial parts of Hypericum species, we applied and modified BP formulae to calculate the anticancer potential (AP) of methanol–water microextracts (MW) from H. canariense (CMW), H. grandifolium (GMW) and H. glandulosum (LMW) (Equation (5)). From the biological tests and regarding GI50 values, the MW microextract from H. reflexum (RMW) was not subjected to the chemical analysis by LC–MS, and consequently it was considered the sample with the less AP.

(5) $$Anticancer\;Potential\;\left(AP\right)=\left(N+1\right)\;\times \;Total\;Activity\;\;\times \;\frac{C_{total}}{C_{known}}\;$$

**Figure 6:**
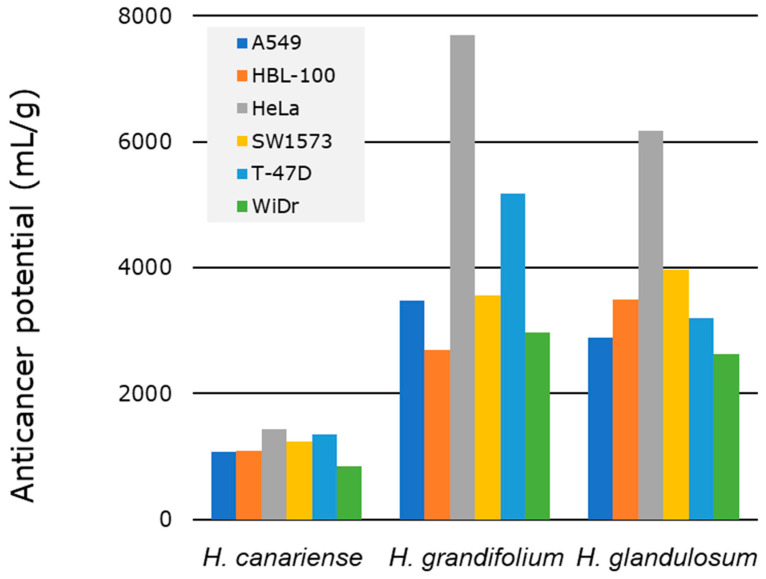
Anticancer potential of MW microextracts from *Hypericum* species.

The anticancer potential values from the MW were obtained based on one primary and two secondary bioassays related to anticancer activity (Figure 6). From the first biological test we conclude that none of the MW microextracts were the most active one. Finally, GMW was selected as the most active one in two of the secondary bioassays: cell migration disturbances in A549 cells and formation of acidic vacuoles in HeLa cells. Table 3 shows the *N* and chemical novelty values considered for the calculation of the AP of MW microextracts from *H. grandifolium*, *H. glandulosum* and *H. canariense*. The TA for these MW microextracts were computed in Section 2.2.2 (Table S2). Overall, the AP values permit to sort the microextracts: GMW > LMW> CMW (*H. grandifolium* > *H. glandulosum* > *H. canariense*). These results also allow select *H. grandifolium* Choisy as the species with the highest AP as a source of polar potentially bioactive compounds. 

### 2.5. MW Microextracts of H. Grandifolium Induce Cell Death in HeLa Cells

Based on this, we selected the MW microextract of *H. grandifolium* (GMW) to run a more detailed study on HeLa cells using label-free continuous live-cell imaging. Live-cell imaging opens a new window in the study of cell response to potential bioactive compounds. The ability to evaluate the behavior of a population at single cell level facilitates the study of phenotypic changes between subgroups of individuals. Moreover, live imaging enables checking the effects at continuous time points, changing the concept of dose–response relationship at fixed times.

**Figure 7:**
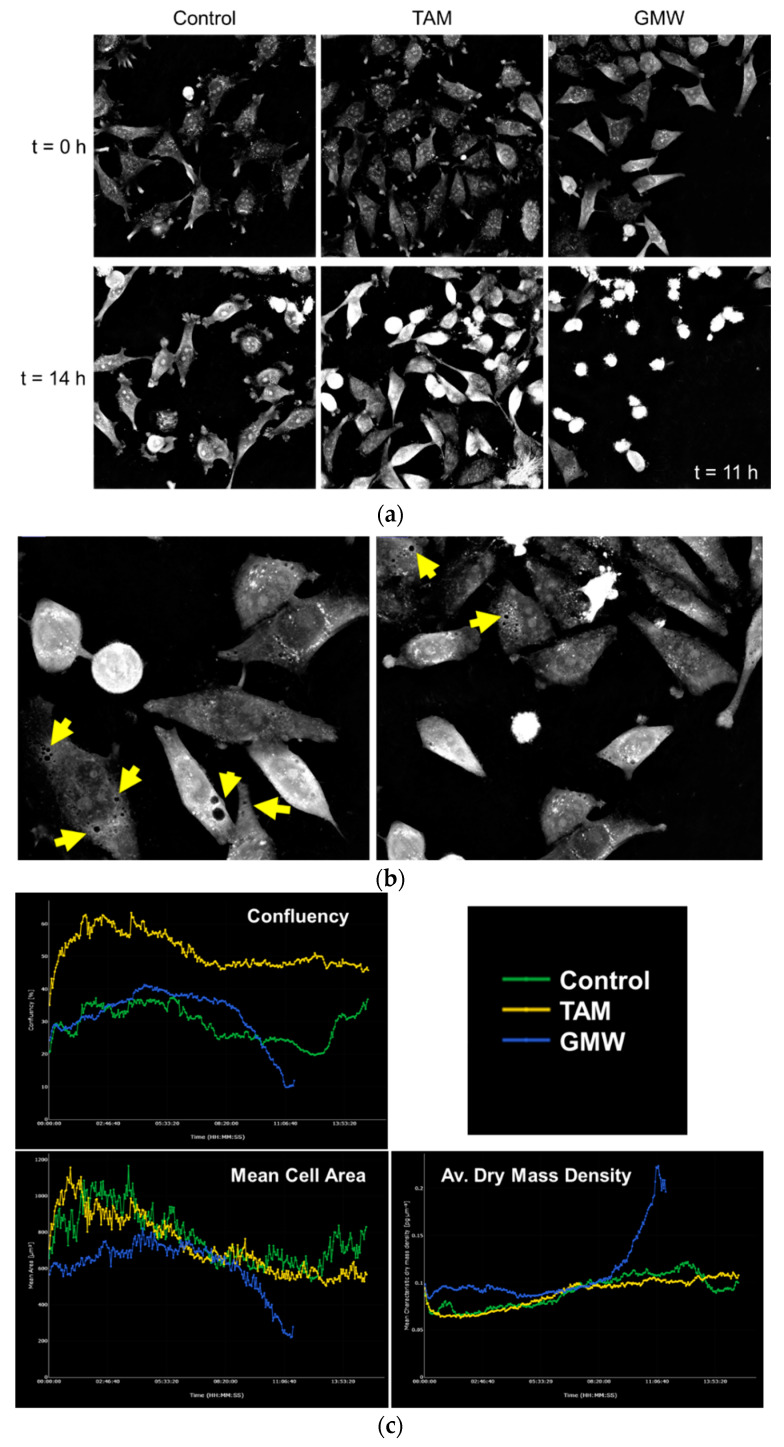
Live-cell imaging study on HeLa cells. (**a**) Representative images of the experiments for untreated (control) cells and cells exposed to 10 µM TAM or 100 µg/mL GMW. (**b**) Images of cell vacuolization after treatment with GMW for 5 h. Yellow arrows indicate cytoplasmic vacuoles observed. (**c**) Confluency, Mean Cell Area and Average Dry Mass Density obtained with STEVE software based on refractive indices resulting from CX-A observation over time. Green: untreated cells. Yellow: TAM (10 µM). Blue: GMW (100 µg/mL).

HeLa cells were exposed to GMW and the effects were monitored for 15 h. For comparison purposes, we used TAM. Figure 7 depicts how GMW is able to induce death to HeLa cells earlier and in extended populations than TAM (Figure 7a). In consonance with AO staining results (Figure 5), cells are highly vacuolated (Figure 7b) in the GMW-treated group. This feature could be induced not only by the pharmacological activity of the extract but as a cell response to an external agent, trying to isolate the exogenous substances. Since live imaging has enabled us to observe cell death progressively, we can differentiate several apoptotic hallmarks. Treatment with GMW induced nuclear condensation and cell shrinkage after 10 h of incubation in a broader way than TAM. For the same time period, cell death was not observed in the control group (Figure 7a, Supplementary material V1). The observed effects were analyzed and quantified with STEVE software (Figure 7c, Figures S2–S4). Cell death is represented as a drastic decrease in the percentage of confluence in the GMW-treated samples. The formation of apoptotic bodies by cell shrinkage is depicted in the diminished cell area. In agreement with this last result, dry mass density is increased in the GMW-treated cells when compared to the control, reflecting the collapsing of cells caused by the extract.

## 3. Materials and Methods

### 3.1. General Experimental Procedures

Acetonitrile, methanol, and formic acid for UHPLC were purchased from Merck (Darmstadt, Germany). Water for UHPLC was purified with a Millipore Simplicity System (Bedford, MA, USA). Solvents for the extraction of plant material were of analytical grade.

### 3.2. Plant Material

The aerial parts of *H. canariense*, *H. grandifolium*, and *H. reflexum* (6 to 8 kg of fresh material) were collected in La Palma and Tenerife (Canary Islands, Spain), during the period between June and July 2019. 7 kg of the aerial parts of *H. glandulosum*, were collected in La Palma and Tenerife (Canary Islands, Spain), in June 2021. All the specimens were collected and identified by Dr. Pedro Luis Pérez de Paz, University of La Laguna. A voucher specimen of species was deposited at TFC Herbarium, University of La Laguna (Tenerife, Spain) (Table 4). Plant material was dried in airy oven at 38 °C temperature for one week. Finally, plant material was stored at room temperature in the darkness.

### 3.3. Extraction of Plant Material

Ground aerial parts (10 g) from *H. canariense*, *H. glandulosum*, *H. grandifolium*, and *H. reflexum*, were sequentially extracted with the same volume (200 mL) of methanol–water (1:1, v/v, MW), methanol (MM), and dichloromethane–methanol (1:1, v/v, DM) for 24 h at room temperature. The resulting extracts were filtered and concentrated under reduced pressure to evaporate the less volatile solvent. All extracts were resuspended in water and lyophilized affording MW, MM and DM extracts, respectively. Thereafter, the microextracts were weighed and stored at 4 °C until further processing.

### 3.4. UHPLC–DAD–MS3 Analysis

UHPLC–DAD–MS analyses of raw and aqueous extracts were conducted using a previously established method [67] with some modifications. Thus, 10 mg of polar microextracts (CMW, GMW and LMW) were dissolved in 1 mL of methanol–water (1:1, v/v) and subjected to UHPLC analyses. The separation was carried out on a Kinetex XB-C_18_ (150 mm × 2.1 mm × 1.7 μm, Phenomenex, Torrance, CA, USA). The mobile phases were 0.1% formic acid in water (A), and 0.1% formic acid in acetonitrile (B), and elution was conducted with the following gradient: 0 min–3% B, 60 min–26% B, 120 min–95% B. The flow rates were 0.3 mL/min. The UV–Vis spectra of the detected compounds were recorded over the 190–600 nm range. The chromatograms of these microextracts) were recorded at 280 nm. Mass spectra were recorded in the positive and negative ion modes. Compounds were characterized based on the maxima observed in their UV–Vis spectra and on their MS spectra. Additionally, the Reaxys database was searched for compounds previously detected and identified in *Hypericum* species.

### 3.5. Cell, Culture and Plating

The human solid tumor cell lines A549 (lung), HBL-100 (breast), HeLa (cervix), SW1573 (non-small-cell lung), T-47D (breast), and WiDr (colon) were used in this study. These cell lines were a kind gift from Prof. Godefridus J. Peters (VU Medical Center, Amsterdam, The Netherlands). Cells were maintained in 25 cm^2^ culture flasks in RPMI 1640 supplemented with 5% FBS and 2 mM L-glutamine in a 37 °C, 5% CO_2_, 95% humidified air incubator. For all tests, exponentially growing cells were trypsinogen and resuspended in an antibiotic-containing medium (100 units penicillin G and 0.1 mg of streptomycin per mL). Single-cell suspensions were counted with Moxi Z. After counting, dilutions were made to give the appropriate cell densities for inoculation onto the appropriate (microtiter) plates.

### 3.6. Antiproliferative Tests

The tests were performed in 96-well plates using the SRB assay [68] with the following specifications. Cell seeding density was 2500 cells/well for A549, HBL-100, HeLa, and SW1573, and 5000 cells/well for T-47D and WiDr. Stock samples were dissolved in DMSO at an initial concentration of 100 mg/mL. Test samples were prepared by decimal serial dilutions of the stock sample to give a final test concentration of 250, 25 and 2.5 µg/mL. The incubation time was 48 h. The optical density of each well was measured at 530 (primary) and 620 nm (secondary) using a microplate reader (Power Wave XS, BioTek, Winooski, VT, USA). The antiproliferative activity, expressed as 50% growth inhibition (GI_50_) values, was calculated according to the NCI formulas [49]. GI_50_ represent approximate values. Following NCI guidelines, the concentrations giving PG values above and below PG = 50 were used to make interpolations on the concentration axis.

The criteria used to categorize the cytotoxicity of the microextracts on the NCI’s protocol was as follows: GI_50_  ≤  20 µg/mL = highly cytotoxic, GI_50_ ranged 21–200 µg/mL = moderately cytotoxic, and GI_50_ > 201 µg/mL = weakly cytotoxic [69].

### 3.7. Cell Migration Assay

To study cell migration, we used the wound healing (scratch) assay [70]. Single-cell suspensions of A549 cells were seeded onto a 24 well plate at a density of 50,000 cells/well. Cells were incubated until they reached >90% confluence. Afterwards, a mark was drawn on the outside bottom of each well. This enabled finding the reference point when taking pictures. For each well, a scratch on the cell culture was made perpendicularly to the mark using a sterile p200 tip. Then, the medium was replaced with fresh medium with 2.5% FBS. This allows us to diminished the interference caused by cell proliferation during the time of assay, evaluating only the cell migration. Pictures were taken with a brightfield microscope (Axiovert 40 CFL, Zeiss, Germany) at one magnification (5X) using the software ZEN 2012 (blue edition v1.1.0.0) (accessed on 10 August 2022) at different time intervals (0, 6 and 24 h since the formation of the scratch). For the quantification of cell migration, TScratch, an image software based in MATLAB, was used adapting threshold of images to measure the area of the wound made [71]. Results are represented as percentage of the closed area as defined by Equation (6).

(6) $$Wound\;Closure\;\%=\left(\frac{A_{t=0}-A_{t=\Delta t}}{A_{t=0}\;}\right)\;\times \;100$$

### 3.8. Acridine Orange Staining Assay

To evaluate the induction of acidic vacuoles by the plant extracts the acridine orange staining assay was used. Briefly, single-cell suspensions of HeLa were seeded onto a 6 well plate at a density of 100,000 cells/well. After 24 h extracts diluted in DMSO were added and incubated for an additional period of 24 h. Then, the medium was removed, cells were detached with trypsin, resuspended with PBS (200 µL), transferred to 1.5 mL tubes and centrifuge 1200 rpm × 5 min (Centrifuge 5418 R, Eppendorf). Each group of cell suspensions were incubated with 1 mL of acridine orange solution (20 µM in PBS) for 30 min at room temperature under dark conditions. After centrifuge at 1200 rpm for 5 min, cells were washed twice with PBS to remove the excess of staining, centrifuged one last time and resuspended in PBS. Then, 100 µL of each cell suspension were added onto a black-walled, clear-bottom, flat bottom 96 well plate and measured fluorescence intensity at 502/528 excitation/emission wavelengths using a microplate reader (Varioskan LUX VLBL0TD0, Thermo Fisher Scientific, Waltham, MA, USA). The number of cells was normalized with crystal violet staining and suspending the dye with 1% SDS in PBS, and finally measuring the optical density at 595 nm using a microplate reader as aforementioned.

For fluorescence microscopy images, cells were seeded onto a coverslip into a p6 well at the aforementioned seeding density. Treatments were made exactly the same as previously described and then staining was performed directly on the wells after removal of the medium. Two washes with PBS were made and coverslips were mounted fresh over glass slides to observe under fluorescence microscopy using SP5 Leica apparatus with LAS-AF software (Leica Microsystems) equipped with I3 (450–490) and N2.1 (515–560) excitation filters for green and red channels, respectively. Both channels were merged into individual images using ImageJ. 

### 3.9. Live-Cell Imaging

A CX-A imaging platform microscope (Nanolive SA, Lausanne, Switzerland) was used to measure refractive indices, creating a holotomographic 3D image of the cells. HeLa cells were cultured at a density of 50,000 cells/well onto a IBIDI μ-Dish, 35 mm high (Martinsried, IBIDI, Germany) and treated with or without 100 µg/mL of extract right before the acquisition of the images. Data obtained were transferred to FIJI (NIH, USA) for image analysis. STEVE software (Nanolive SA) was used for the analysis of the refractive indices and obtention of the Confluency, Mean Cell Area, and Mean Average Dry Mass Density.

## 4. Conclusions

In summary, we have proposed a general methodology and empirical equation to assess the BP during the screening of extracts from plants. We applied the said protocol to four *Hypericum* species from the Canary Islands (*H. canariense* L., *H. glandulosum* Aiton, *H. grandifolium* Choisy and *H. reflexum* L.f.), specifically to evaluate the anticancer potential (AP) of their methanol–water microextracts. The assessment of AP involved the evaluation of the yield of extraction, the assessment of the antiproliferative activity of the microextracts and their dereplication by LC–MS. After the application of this pharmacognostic protocol, it was possible to assess the AP of the methanol–water microextracts and to select *H. grandifolium* as the best candidate to carry out the bioguided isolation of polar natural products with high added value. 

## Figures and Tables

**Table 1:** Yield of microextracts from *Hypericum* species from the Canary Islands.

	Yield (%)			
Species	MW	MM	DM	Total
C	8.5	9.5	2.4	20.4
G	7.5	8.0	1.9	17.4
L	13.9	7.6	9.1	30.6
R	14.3	6.9	1.2	22.1

C = *H. canariense*; G = *H. grandifolium*; L = *H. glandulosum*; R = *H. reflexum*; MW = methanol/water microextracts; MM = methanol microextracts; DM = dichloromethane/methanol microextracts.

**Table 2:** Qualitative analysis of polar microextracts by liquid chromatography photodiode array detection mass spectrometry (LC–PDA–MSn).

Peak	RT (min)	(max)	MS [M+H]^+^	MS [M-H]^−^	CMW	GMW	LMW	Putative Compound
1	3.3	269	171.03	169.03	-	✔	-	gallic acid [59]
2	6.6	259, 293	154.99	153.07	✔	✔	-	3,4-dihydroxybenzoic acid [60]
3	7.6	214, 256	205.30	203.18	-	-	✔	unidentified compound
4	7.8	202, 279	-	315.22	✔	-	-	unidentified compound
5	8.1	205, 262, 294	343.19	341.18	-	✔	-	unidentified compound
6	11.3	214, 325	355.2	353.25	✔	✔	✔	neochlorogenic acid [61]
7	12.9	287, 316	343.21	341.28	-	-	✔	unidentified compound
8	14.4	209, 311	339.27	337.27	-	✔	✔	*p*-coumaroylquinic acid isomer [62,63]
9	15.4	217, 310	339.20	337.21	✔	✔	✔	*p*-coumaroylquinic acid isomer [62,63]
10	16.5	279	579.19	577.19	✔	-	-	dimeric procyanidin type B [64]
11	17.9	216, 325	355.20	353.25	✔	-	✔	chlorogenic acid [42]
12	20.0	214, 325	355.19	353.24	-	-	✔	cryptochlorogenic acid [63]
13	21.7	280	579.20	577.19	✔	-	-	dimeric procyanidin type B [64]
14	23.3	279	579.17	577.19	✔	✔	✔	dimeric procyanidin type B
15	24.6	279	291.13	289.17	✔	✔	✔	catechin [64]
16	25.9	203, 257, 318, 369	423.19	421.16	✔	-	-	mangiferin/isomangiferin [63]
17	27.2	203	407.18	405.28	-	✔	-	neolancerin/xanthohypericosider [60]
18	29.2	279	865.23	863.23	-	✔	-	unidentified procyanidin
19	30.2	279	867.24	865.26	✔	✔	✔	trimeric procyanidin type C [64]
20	33.2	279	-	576.17 ^a^	✔	✔	✔	tetrameric procyanidin B type [64]
21	35.9	279	-	720.22 ^b^	-	✔	✔	pentameric procyanidn B type [64]
22	37.6	209, 255, 354	611.21	609.20	-	-	✔	rutin [19,42]
23	38.5	256, 353	465.18	463.14	✔	-	✔	quercetin 3-*O*-glucoside [63]
24	39.8	203, 255, 353	479.16	477.18	✔	-	-	quercetin 3-*O*-glucuronide [63]
25	41.4	286	-	361.17	✔	-	-	unidentified compound
26	42.4	203, 265, 344	595.20	593.19	-	-	✔	kaempferol 3-*O*-rhamnoglucoside
27	43.7	203, 255, 350	449.19	447.17	-	✔	-	quercetin 3-*O*-rhamnoside [19,42]
28	55.5	203, 254, 368	303.11	301.1	✔	✔	✔	quercetin [19,42]
29	60.0	217, 310, 353	611.17	609.20	✔	✔	-	quercetin *O*-*p*-coumaroylhexoside [63]
30	65.1	218, 368	287.10	285.08	-	-	✔	kaempferol [19]
31	67.7	219, 342	749.16	747.18	-	✔	-	unidentified compound
32	68.4	265, 340	749.15	747.17	-	✔	✔	unidentified compound
33	71.1	268, 335	539.16	537.30	✔	✔	✔	amentoflavone [19]
34	78.8	284, 369, 483, 565	367.34	365.30	-	-	✔	unidentified compound
35	79.2	287, 369, 483	381.32	379.36	-	-	✔	unidentified compound
36	86.7	221, 291	363.32	361.32	-	-	✔	unidentified compound
37	87.4	287, 369	349.31	347.33	-	-	✔	unidentified compound
38	91.9	221, 291	363.35	361.35	-	-	✔	unidentified compound
39	97.3	221, 290	333.31	331.27	-	-	✔	unidentified compound
40	100.0	221, 291	347.33	345.33	-	-	✔	unidentified compound
41	101.0	221, 287	333.34	331.34	-	-	✔	unidentified compound

**^a^** [M-2H]^2−^. **^b^** [M-2H]^2−^.

**Table 3:** Anticancer potential for the most bioactive MW *Hypericum* extracts.

		Chemical Novelty			Anticancer Potential (mL/g)					
Microextracts	*N*	*C_total_*	*C_known_*	*C_total_*/*C_known_*	A549	HBL-100	HeLa	SW1573	T-47D	WiDr
CMW	0	18	16	1.125	1074	1087	1427	1242	1347	846
GMW	2	19	15	1.266	3475	2689	7699	3559	5180	2966
LMW	0	27	16	1.688	2897	3503	6175	3977	3207	2637

**Table 4:** Collection data of four *Hypericum* species.

Plant Species	Common Name	Collection Site	Voucher
*Hypericum canariense* L.	granadillo	Breña Baja. Las Ledas (La Palma island)	53,351
*Hypericum glandulosum* Aiton	malfurada del monte	In between Mirador de Gallegos and Roque Faro (La Palma island)	53,775
*Hypericum grandifolium* Choisy	malfurada	Villa de Mazo. La Tablada (La Palma island)	53,350
*Hypericum reflexum* L.f	cruzadilla	Arico-Granadilla (Tenerife island)	53,346

## Data Availability

The data presented in this study are available on request from the corresponding author. The data are not publicly available due to privacy or ethical restrictions.

## Supplementary Materials

The following supporting information can be downloaded at: https://www.mdpi.com/article/10.3390/molecules27186101/s1, Figure S1: LC–PDA–MSn chromatograms for *Hypericum* MW microextracts; Figure S2: Confluency obtained with STEVE software based on refractive indexes resulting from CX-A observation over time; Figure S3: Mean Cell Area obtained with STEVE software based on refractive indexes resulting from CX-A observation over time; Figure S4: Average dry Mass Density obtained with STEVE software based on refractive indexes resulting from CX-A observation over time; Table S1: Antiproliferative activity of *Hypericum* microextracts; Table S2: Total activity of *Hypericum* microextracts; Video S1: Live-cell imaging of MW microextract of *H. grandifolium*.
