# Supplementary material for: Bioactive Potential: A Pharmacognostic Definition through the Screening of Four Hypericum Species from the Canary Islands
Source: Molecules. 2022 Sep 18;27(18):6101. doi: 10.3390/molecules27186101 (PMC9505652; doi:10.3390/molecules27186101)
Supplement: Supplementary file 1 [file molecules-27-06101-s001.zip › Supplementary Materials.pdf]

Bioactive Potential: A Pharmacognostic Definition through the Screening of Four *Hypericum* Species from the Canary Islands

Rodney Lacret [1,2,*], Adrián Puerta [1], Sebastian Granica [3], Aday González-Bakker [1], Danela Hevia [1], Yiling Teng [1], Candelaria C. Sánchez-Mateo [2], Pedro Luis Pérez de Paz [4] and José M. Padrón [1]

[1] BioLab, Instituto Universitario de Bio-Orgánica Antonio González (IUBO-AG), Universidad de La Laguna, Avda. Astrofísico Francisco Sánchez 2, 38206 La Laguna, Spain
[2] Departamento de Medicina Física y Farmacología, Facultad de Farmacia, Universidad de La Laguna, Tenerife, 38200 La Laguna, Spain
[3] Microbiota Lab, Centre of Preclinical Studies, Medical University of Warsaw, Banacha 1b, 02-097 Warsaw, Poland
[4] Departamento de Botánica, Ecología y Fisiología Vegetal, Facultad de Farmacia, Universidad de La Laguna, Tenerife, 38200 La Laguna, Spain
* Correspondence: rlacretp@ull.es

Table of Contents

Table S1. Antiproliferative activity of *Hypericum* microextracts 2
Table S2. Total activity of *Hypericum* microextracts...2
Figure S1. LC–PDA–MSn chromatograms for *Hypericum* MW microextracts 3
Figure S2. Confluency obtained with STEVE software based on refractive indexes resulting from CX-A observation over time..4
Figure S3. Mean Cell Area obtained with STEVE software based on refractive indexes resulting from CX-A observation over time..5
Figure S4. Average dry Mass Density obtained with STEVE software based on refractive indexes resulting from CX-A observation over time..6

Table S1. Antiproliferative activity (GI_{50}) against six human solid tumor cell lines of extracts from the aerial parts of *Hypericum* species.

Microextracts	Cell lines (*origin*)					
	A549 (*lung*)	HBL-100 (*breast*)	HeLa (*cervix*)	SW1573 (*lung*)	T-47D (*breast*)	WiDr (*colon*)
**CMW**	89	88	67	77	71	113
**CMM**	51	51	25	32	29	79
**CDM**	5.8	7.7	5.4	5.6	7.3	8.2
**GMW**	82	106	37	80	55	96
**GMM**	5.9	4.0	2.7	3.3	4.1	7.7
**GDM**	5.4	6.0	6.3	5.3	7.7	13
**LMW**	81	67	38	59	73	87
**LMM**	41	43	37	36	55	57
**LDM**	41	27	11	13	42	34
**RMW**	140	>250	73	126	86	156
**RMM**	76	74	49	51	59	105
**RDM**	34	61	20	13	46	101

Table S2. Total activity (mL/g) of microextracts from *Hypericum* species against human solid tumor cell lines.

Microextracts	Total activity (mL/g)					
	Cell lines (*origin*)					
	A549 (*lung*)	HBL-100 (*breast*)	HeLa (*cervix*)	SW1573 (*lung*)	T-47D (*breast*)	WiDr (*colon*)
CMW	955	966	1268	1104	1197	752
CMM	1863	1863	3800	2969	3276	1203
CDM	4130	3117	4449	4286	3287	2927
H. canariense	**6948**	**5946**	**9517**	**8359**	**7760**	**4882**
GMW	915	708	2027	937	1364	781
GMM	13556	20000	29630	24242	19512	10390
GDM	3519	3166	3016	3585	2468	1462
H. grandifolium	**17990**	**23874**	**34673**	**28764**	**23344**	**12633**
LMW	1716	2075	3658	2356	1900	1562
LMM	1854	1767	2054	2111	1382	1333
LDM	2220	3370	8272	7000	2166	2676
H. glandulosum	**5790**	**7212**	**13984**	**11467**	**5448**	**5571**
RMW	1021	572	1917	1135	1662	916
RMM	908	932	1408	1352	1169	657
RDM	353	1967	600	923	260	118
H. reflexum	**2282**	**3471**	**3925**	**3410**	**3091**	**1691**

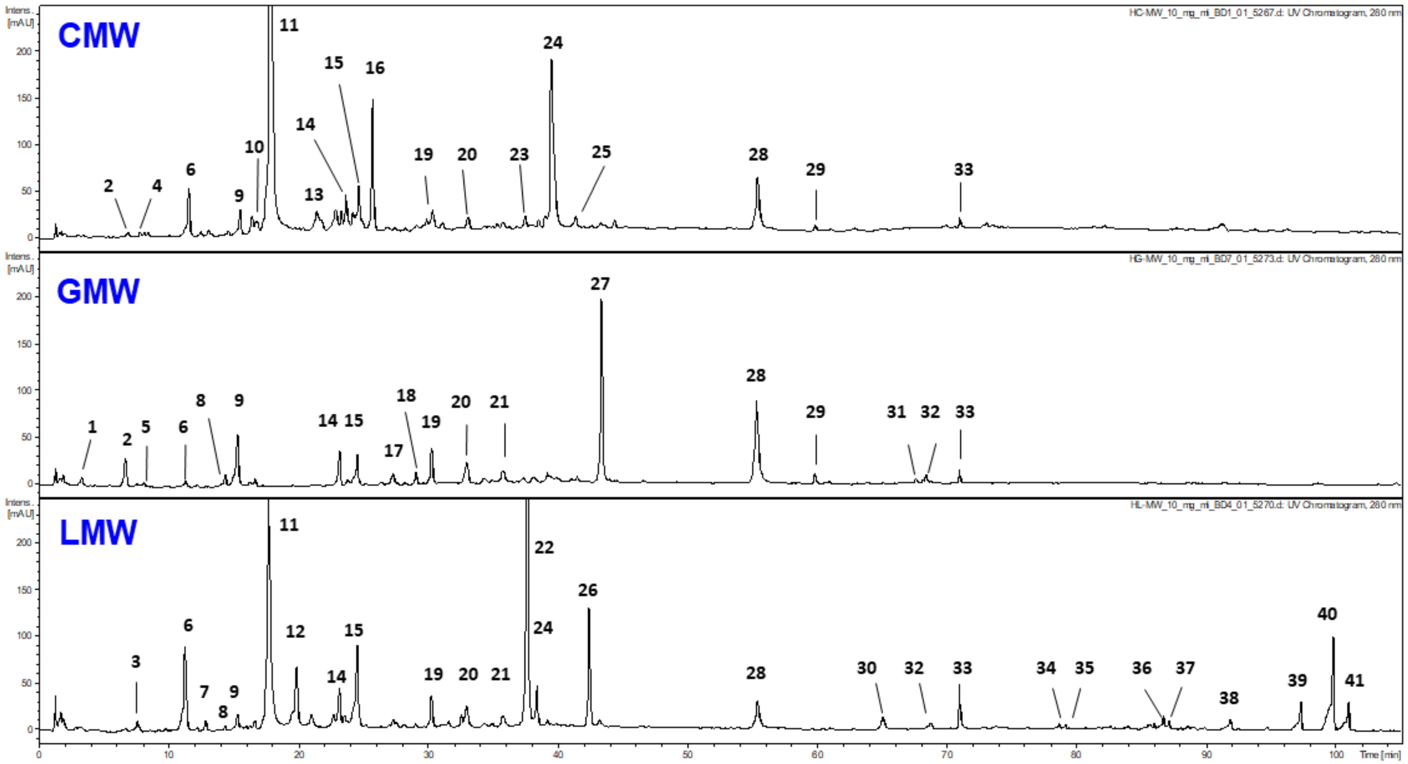

Figure S1. LC–PDA–MSn chromatograms (280 nm) for *Hypericum* MW microextracts.

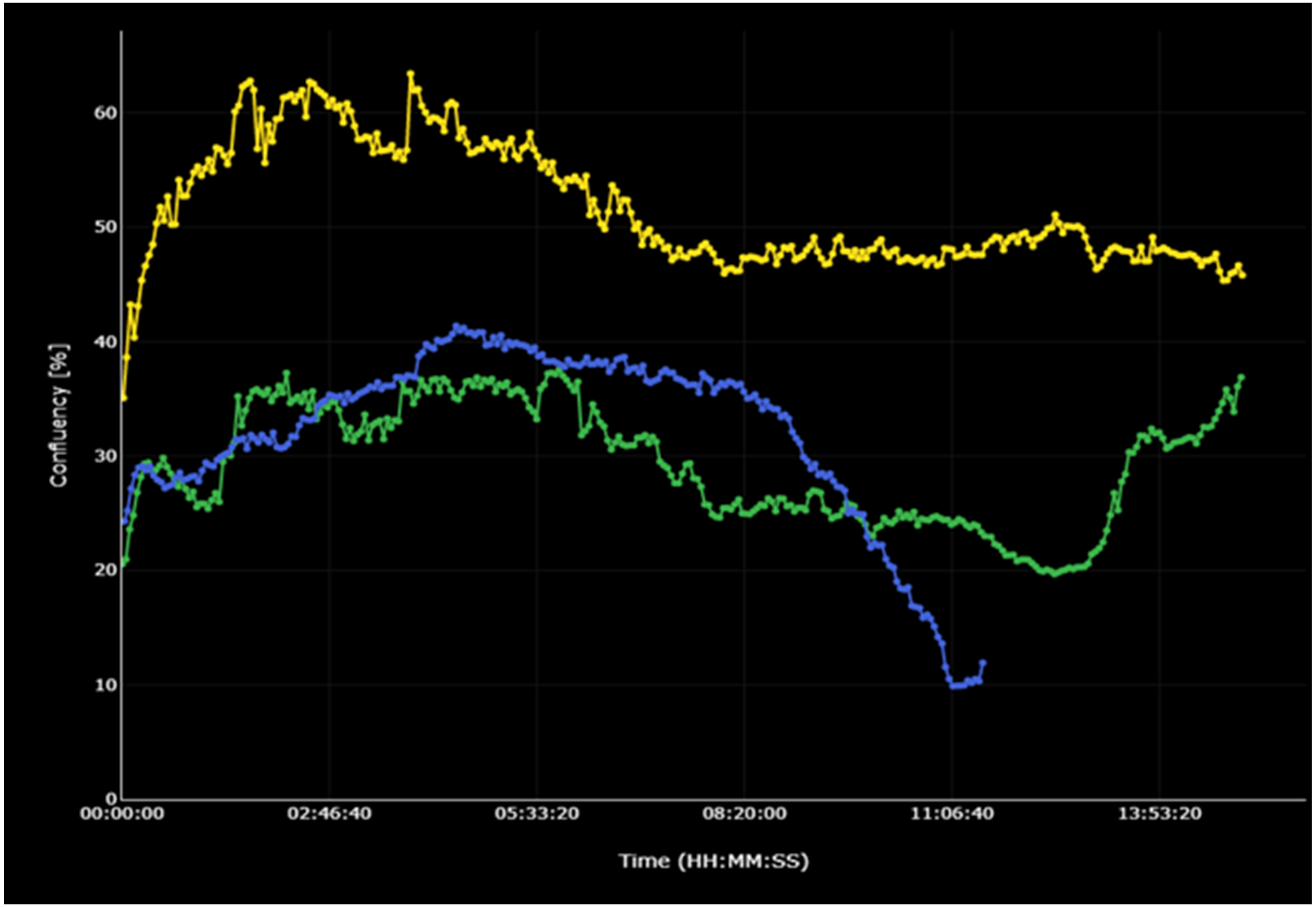

Figure S2. Confluency obtained with STEVE software based on refractive indexes resulting from CX-A observation over time. Green: untreated cells. Yellow: TAM (10 µM). Blue: GMW (100 µg/mL)

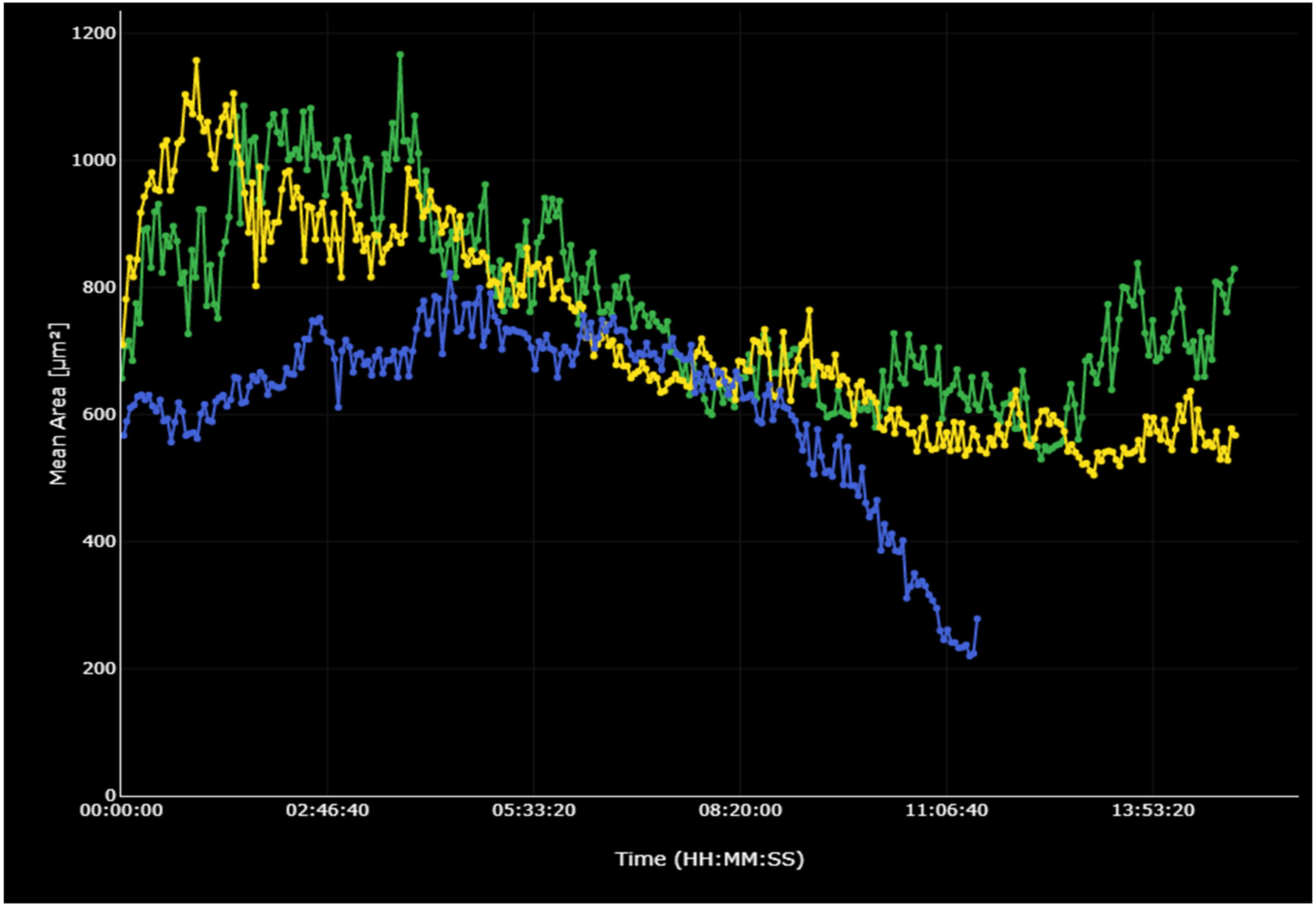

Figure S3. Mean Cell Area obtained with STEVE software based on refractive indexes resulting from CX-A observation over time Green: untreated cells. Yellow: TAM (10 µM). Blue: GMW (100 µg/mL)

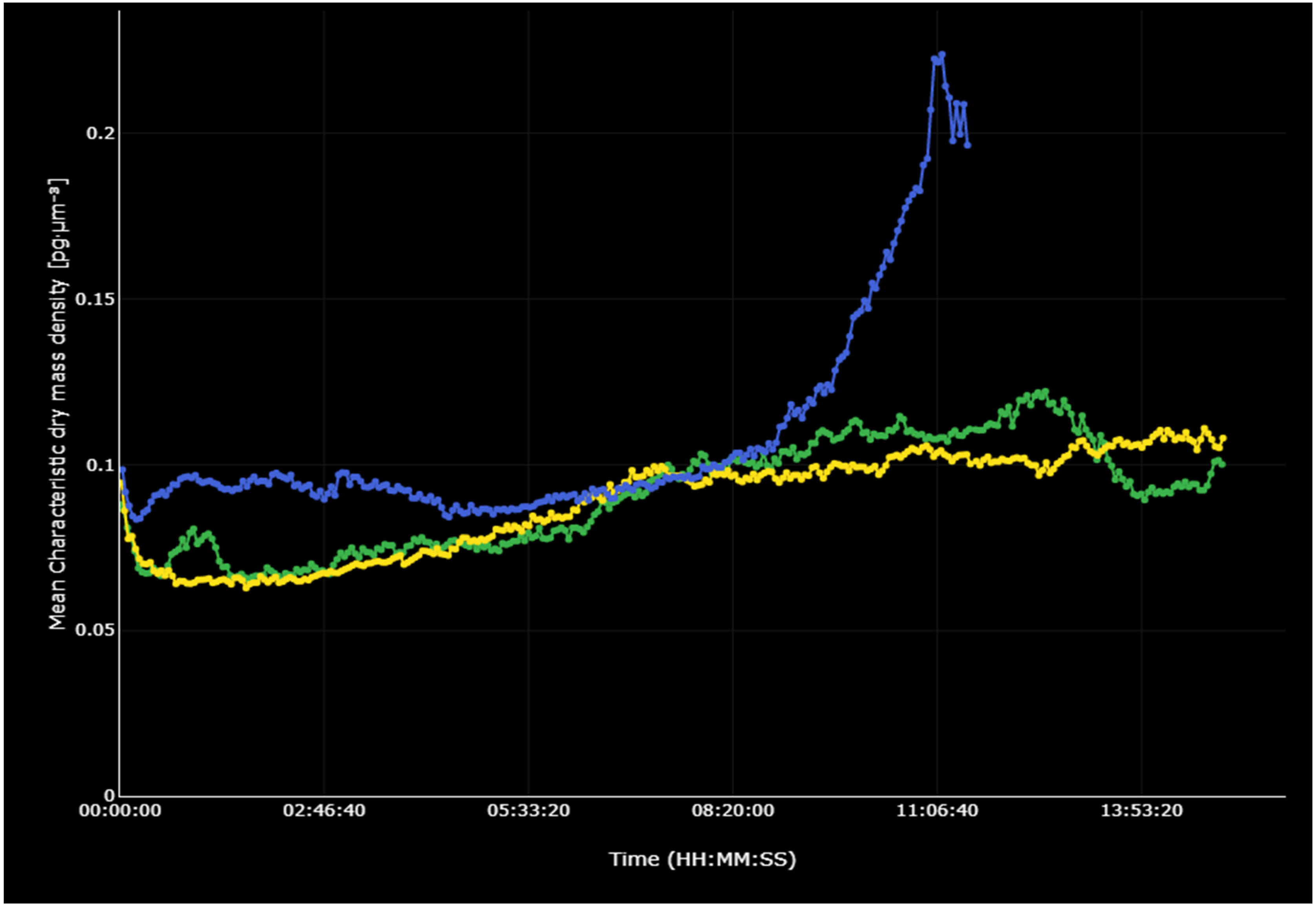

Figure S4. Average dry Mass Density obtained with STEVE software based on refractive indexes resulting from CX-A observation over time. Green: untreated cells. Yellow: TAM (10 µM). Blue: GMW (100 µg/mL).
